# 

**Published:** 1868-11-15

**Authors:** 


### CHICAGO MEDICAL JOURNAL.

J. ADAMS ALLEN, M.D., LL.D., Editor. WALTER HAY, A.M., M.D., Associate Editor.

All letters for the JOURNAL should be addressed to Dr. J. ADAMS ALLEN, No. 71 Dearborn St., Chicago, Ill.

TERMS — $3 Per Annum, in Advance;

$4 if not paid before 1st of July, in each year. Single copies, 25 cents.

### BOOKS AND PAMPHLETS RECEIVED.

FLINT'S PRACTICE OF MEDICINE. Third Edition. H. C. Lea.

MARSHALL'S PHYSIOLOGY. Edited by Francis G. Smith, M.D. H. C. Lea.

MURCHISON ON THE LIVER. William Wood & Co.

RETRUCTIS MYCTALOPICA. By Prof. Dr. Arlt, of Vienna. Lindsay & Blakiston.

CULLEVIER'S ATLAS. Edited by F. G. Bumstead, M.D. Part V., concluding the work.

